# TLR7 Signaling Shapes and Maintains Antibody Diversity Upon Virus-Like Particle Immunization

**DOI:** 10.3389/fimmu.2021.827256

**Published:** 2022-01-19

**Authors:** Xinyue Chang, Pascal Krenger, Caroline C. Krueger, Lisha Zha, Jiami Han, Alexander Yermanos, Salony Roongta, Mona O. Mohsen, Annette Oxenius, Monique Vogel, Martin F. Bachmann

**Affiliations:** ^1^ Department of Rheumatology & Immunology, University Hospital Bern, Bern, Switzerland; ^2^ Department of BioMedical Research, University of Bern, Bern, Switzerland; ^3^ International Immunology Centre, Anhui Agricultural University, Hefei, China; ^4^ Department of Biosystems Science and Engineering, Eidgenössische Technische Hochschule (ETH), Zürich, Basel, Switzerland; ^5^ Institute of Microbiology, Eidgenössische Technische Hochschule Zürich, Zurich, Switzerland; ^6^ Department of Pathology and Immunology, University of Geneva, Geneva, Switzerland; ^7^ Jenner Institute, University of Oxford, Oxford, United Kingdom

**Keywords:** Qβ-VLP, Fel d 1, TLR7, B cell receptor repertoire, hypermutation

## Abstract

Virus-like particles (VLPs) are used in different marketed vaccines and are able to induce potent antibody responses. The innate pattern recognition receptors TLR7/8 recognize single stranded (ss) RNA naturally packaged into some VLPs and have been shown to enhance the production of IgG antibodies upon immunization. Here we demonstrate that, upon immunization with RNA-loaded bacteriophage-derived VLP Qβ, TLR7 signaling accelerates germinal center formation, promotes affinity/avidity maturation of VLP-specific IgG and isotype switching to IgG2b/2c. These findings extrapolated to antigens displayed on Qβ; as Fel d 1, the major cat allergen, chemically attached to Qβ also induced higher affinity/avidity IgG2b/2c antibodies in a TLR7-dependent fashion. Chimeric mice lacking TLR7-expression exclusively in B cells demonstrated that the enhanced IgG responses were driven by a B cell intrinsic mechanism. Importantly, deep sequencing of the BCR repertoire of antigen-specific B cells demonstrated higher diversity in mice with TLR7 signaling in B cells, suggesting that TLR7-signaling drives BCR repertoire development and diversity. Furthermore, the current data demonstrate that high levels of clonal diversity are reached early in the response and maintained by TLR7 signaling. In conclusion, TLR7 signaling enhances levels and quality of IgG antibodies, and this finding has major implications for vaccine design.

## Introduction

Virus-like particles (VLPs) are known to be an excellent vaccine platform and several VLP-based vaccines have been commercially available for decades (1, 2). In addition to an excellent safety profile, potent immunogenicity grants VLPs as attractive vaccine candidates due to their highly repetitive and organized surface structures, as well as the viral size (20-200nm) that allows free drainage of VLPs into lymph nodes (3). Furthermore, it has been demonstrated that optimal spacing of 5-10 nm between the VLP surface antigens might be particularly beneficial for the induction of effective antiviral antibodies. As shown recently, such spacing is sometimes not present in native viruses as e.g. SARS-CoV-2 (4), but can easily be achieved with recombinant VLPs (5–7).

The innate immune system does not only recognize the repetitive viral-like structures as a pathogen-associated structural pattern (PASP) (8), but also senses the pathogen-associated molecular patterns (PAMPs), such as nucleic acids including ssRNA, located in endosomes instead of the cytosol. Other examples of PAMPs include dsRNA and unmethylated CpG, which rarely appear in mammalian hosts but are unique and abundant in microbes (9). The corresponding cellular pattern recognition receptors (PRRs) are present in different cellular locations, and some even extracellular in blood and tissue fluids to monitor pathogens everywhere. Of all PRRs, the Toll-like receptor (TLR) family, which consists of more than ten members, takes a key role in pathogen recognition. Each receptor detects a different PAMP, such as TLR4, TLR7/8 and TLR9 sensing LPS, ssRNA and DNA rich in unmethylated CpG, respectively (10). Once they encounter the ligands, the TLRs dimerize and initiate signaling cascades, leading to production of immune effector molecules (11). One of the core adaptor molecules of TLR signaling is MyD88, which interacts with the dimerized TLRs and recruits downstream signaling molecules such as TRAF6. Furthermore, MyD88 signaling in B cells has been shown to enhance T cell-dependent and -independent antibody responses upon VLP immunization (12). Besides the contributions of TLRs in innate immune profiles, more and more evidence shows that the B cell-intrinsic TLR signaling supports the production of antigen-specific antibody responses, providing a key link between the innate and adaptive immune system (13–16).

Long-lasting, high affinity antibody responses are mainly accomplished by differentiated B cells that have gone through germinal center (GC) reactions with the help of follicular T helper cells in secondary lymphoid organs. The importance of TLR7 signaling in driving the GC reaction was reported e.g. by J. Bessa et al., showing that the presence of B cells-intrinsic TLR7 signaling could compensate an impaired GC reaction caused by lack of IL-21 signaling (17). A similar study on B cell receptor (BCR) repertoires stated that cooperation with TLR7 signaling promoted antibody affinity of GC B cells (18). Moreover, IgG subclass 2a/2c, 2b antibodies in mice, which have considerably enhanced effector function compared to other IgG subclasses, are preferably induced upon TLR7 stimulation (19, 20). Indeed, IgG2a/2c and IgG2b isotype antibodies were shown to exhibit enhanced protective function in viral (21), bacterial (22) and parasite infections (23). Hence, the prokaryotic ssRNA that some VLPs spontaneously encapsulate during production in *E. coli (* 24), further enhances their immunogenicity by triggering TLR7 signaling.

The bacteriophage-derived Qβ VLPs has been extensively studied as a vaccine delivery platform that elicits potent primary and secondary antibody responses. In addition, Qβ has also been used for displaying foreign antigens to generate prophylactic and therapeutic vaccines of choice. As a recent example, a therapeutic Qβ-based VLP displaying islet amyloid polypeptide (IAPP) on its surface was able to induce potent antibodies against IAPP aggregates in islets of vaccinated mice, causing a delayed onset of type II diabetes in this murine disease model (25). However, the molecular basis underlying this enhanced response against antigens displayed on RNA-loaded VLPs is still ill understood.

In this study, we used Fel d 1 as a model antigen. Fel d 1 is the major allergen in cats, accounting for >90% reactivity in cat allergic individuals (26). We have previously screened three anti-Fel d 1 monoclonal antibodies (mAbs) from Qβ-Fel d 1 immunized mice *via* mammalian cell display (27, 28). These mAbs recognize different epitopes of Fel d 1, forming a well-defined system to investigate the mechanism of allergic sensitization and desensitization (28–30). With this background knowledge, we used Fel d 1 as the model antigen displayed by Qβ.

In the present work, we aimed to examine at the molecular level the contribution of TLR7 signaling to antibody responses against Fel d 1 displayed on RNA-loaded Qβ. Fel d 1-specific antibody responses were assessed in wild type and TLR7 deficient mice after Qβ-Fel d 1 immunization. Our results demonstrate that lack of TLR7 signaling significantly dampened Fel d 1-specific IgG responses, in particular the ones of IgG2b and 2c isotypes. Furthermore, similar observations were made in a mouse model where TLR7 is exclusively deficient in B cells. In addition, the Fel d 1-specific BCR repertories demonstrated maintenance of higher diversity in immunized mice with intact TLR7 signaling. Among all Fel d 1-specific IgGs, we found that the VH-gene usage was strongly biased to IGHV 1-7, the V_H_ gene family that was also used by one of the monoclonal antibodies against Fel d 1 we previously generated. Thus, this study identifies an important role of TLR7 signaling in driving and shaping specific antibody responses against VLPs and antigens displayed on them.

## Materials and Methods

### Qβ-VLP, Fel d 1 Antigen Expression and Qβ-Feld 1 Production

The self-assembled Qβ-VLP was produced in *E. coli*, and purification was as described previously (31). As the prokaryotic RNA was spontaneously packed during particle assembly, the RNA was removed using RNase A to obtain empty Qβ particles (20). Briefly, Qβ was buffer exchanged to 20 mM HEPES buffer with 100 kD Amicon Ultra Filters (MERCK, cat. UFC510024) and then incubated with 10 mg/ml RNase A at 37˚C for 6 hours. The clearance was confirmed by the absence of RNA band on 1% agarose gel.

The antigen Fel d 1 was expressed in *E. coli* as described elsewhere (28). In short, the pure Fel d 1 was obtained after purifying the bacterial lysis through nickel column (His Trap HP, Cytiva, cat. 17524802) and size exclusion column (HiLoad 26/600 Superdex 75pg, GE Healthcare, cat. 28-9893-34).

Then the purified Fel d 1 was used for chemical coupling to Qβ with Succinimidyl 6-((beta-maleimidopropionamido) hexanoate) (SMPH, ThermoFisher Scientific, cat. 22363) linker. Firstly, Qβ-VLP was incubated with SMPH at 25°C for 30 min with 400 rpm shaking, and Fel d 1 was incubated with mild reducing agent Tris-(2-Carboxyethyl) phosphine (TCEP, Invitrogen, T2556) at same condition. Then the excess SMPH was removed by passing through 7 kDa Zeba Spin Desalting column (Thermo Scientific, cat. 89882), after which Qβ-SMPH and reduced Fel d 1 was mixed and incubated for 3 h, 25°C, 400 rpm shaking. Afterwards, the coupled Qβ-Fel d 1 was centrifuged through 100kD Amicon Ultra Filters to get rid of free Fel d 1. The coupling efficiency was estimated as 20%-30%, according to methods reported before (32).

### Mice Immunization

*C57BL/6JRccHsd* wild type mice were purchased from Envigo (The Netherlands) at age of 7 weeks; TLR7 knock-out (B6.129P2-Tlr7tm1Aki) and JH knock-out mice were kindly donated from Prof. Dr. Pål Johansen and Prof. Andrew Macpherson, respectively. All mice were kept in specific pathogen-free (SPF) facility of DBMR in Bern, and all experiments were performed in accordance with ethical principles and guidelines of the Cantonal Veterinary Office Bern, Switzerland. The construction of bone marrow chimeras was referred to C. Krueger et al. (19). And all immunization was performed by subcutaneous injection of 30 μg Qβ-Fel d 1 on female mice (8-12 weeks old), from which serum samples were obtained by tail vein bleeding at indicated time points.

### Flow Cytometry

Spleens of immunized mice were harvested at indicated time points and single cell suspensions were obtained by passing through 70 μm cell strainer (Greiner Bio-ONE, cat. 542070). Single cells were suspended in FACS buffer (2% FBS in PBS) and incubated with antibody at 4°C. To distinguish the Qβ-specific B cells in germinal center, cells were firstly stained with Qβ-AlexaFluoro 488 and PNAbio (Vector Laboratories, cat. B-1075), and then Fc-receptors were blocked with anti-mouse CD16/CD32 (BD Bioscience, cat. 553142). Finally, streptavidin-APC Cy7 (BD Bioscience, cat. 554063) and anti-mouse CD38-PerCP Cy5.5 (Biolegend, cat. 102722) were applied to determine germinal center cells, anti-mouse B220-PE Cy7(BD Bioscience, 552772) for B cells, anti-mouse IgM-PE (Jackson ImmunoResearch, cat. 115-116-075), IgD-PE (eBioscience, cat. 12-5993-83), CD4-PE (BD Bioscience, cat. 553653), CD8-PE (BD Bioscience, cat. 553032), CD11b-PE (BD Biosience, cat. 553311), CD11c-PE (BD Biosience, cat. 553802), GR1-PE (BD Biosience, cat. 553128) to exclude other cell types (CD4, CD8: T cells; CD11b: monocytes and macrophages; CD11c: dendritic cells; GR1: neutrophils) and immature B (IgM^+^IgD^+^) cells.

### Fel d 1-Specific B Cells Sorting by Fluorescence-Activated Cell Sorting (FACS)

The Fel d 1-specific B cells from spleens of immunized mice were processed and stained as described above with some adjustments. After obtaining single cell suspension, B cells were enriched using B cell Isolation Kit (Stemcell Technologies, cat. 19854). Fel d 1 displayed on surface of AP205, an unrelated VLP, was labeled with AlexaFluoro 647 and used to amplify the binding magnificence of Fel d 1 to specific B cells. In addition, AlexaFluoro 488 labeled Fel d 1 was used to detect Fel d 1-specific B cells as well. As a result, Fel d 1-specific B cells were selected with double positive of Fel d 1-AlexaFluoro 488 and AP205-Fel d 1-Alexafluoro 647. The detailed gating strategy is illustrated as **Figure S2**.

### Fluorescence Immunohistochemistry Microscope

Spleen tissue was embedded in Tissue-Tec OCT (Sakura, cat. 4583) and kept on dry ice, and then was sectioned to 5 μm slides at -20°C using cryostats (Thermo Scientific). Sections were fixed in cold acetone for 10 min and air dried before prewetting in PBS for 5 min. Afterwards, tissue samples were blocked with 1% BSA and 1% normal mouse serum in PBS for 20 min, after which the PNAbio diluted in 1% BSA and 1% normal mouse serum in PBS was added and incubated for 1 h. Next, slides were washed 3 times with PBS for 5 min and then incubated with secondary antibody solution (Qβ-AlexaFluoro 488, B220-AlexaFluoro 647 and Streptavidin-AlexaFluoro 546) for 45 min in dark. After washing 3 times with PBS, samples were finally mounted with a drop of Fluoromount G solution (Thermo Fisher Scientific, cat. 00-4958-02) and imaged with Zeiss Axio Imager.A2 microscope. Images were captured by using the AxioVision software (ZEISS).

### ELISA and Avidity ELISA

Corning half area 96-well plates were coated with Qβ (1 μg/ml) or Fel d 1 (1 μg/ml) at 4˚C overnight. And then serum samples (from 1:10 dilution) were added and serially diluted in wells and incubated for 1 h at room temperature after blocking. Afterwards, detecting antibody goat anti-mouse IgG-POX (Jackson ImmunoResearch, cat. 115-035-071) was incubated on plates. For IgG1, 2b and 2c isotypes assessment, rat anti-mouse IgG1-HRP (BD Pharmingen, cat. 559626), goat anti-mouse IgG2b-HRP (Invitrogen, cat. M32407), and goat anti-mouse IgG2c-HRP (SouthernBiotech, cat. 107805) were used, respectively. Finally, the plates were developed with TMB substrate, stopped by 1 mol/L sulfuric acid and read at OD450nm in a reader (Molecular Devices, SpectraMax M5).

To determine the quality of antibodies produced by immunized mice, avidity ELISA was performed. Basically, two plates were parallel performed and one was washed with PBS-0.05% Tween-7 M urea for 3 times by 5min incubation, while the other with PBS-0.05% Tween, after mice sera incubation. Avidity index was calculated as ratio of OD (PBS-0.05% Tween-7 M urea) to OD (PBS-0.05% Tween) with same dilution.

### BCR Repertoire Preparation and Analysis

mRNA was extracted from sorted Fel d 1-specific B cells with NucleoSpin RNA XS kit (MACHEREY-NAGEL, cat. 740902.50) and then cDNA was obtained by incubating at 37°C for 1 h according to High-Capacity RNA-to-cDNA Kit protocol (fisher scientific, cat. 4387406). BCR repertoire pool was prepared as protocol described previoulsy (33). Briefly, PCR was performed to get variable fragments of subclasses (IgG1, 2b, 2c subclass primers were listed in **Table S1**) with KAPA HIFI HotStart ReadyMix (Roche, cat. KK2601) as follows: 95°C, 5 min; 40 × (98°C, 30 s; 55.8°C, 30 s; 72°C, 20 s); 72°C, 10 min. And the 400-500bp bands were recycled and analyzed by Fragment Analyzer (Advanced Analytical), which afterwards were attached with sequencing adaptor indexes using Nextera XT Index Kit (Illumina, FC-131-1024). Finally, the sample libraries were pooled and sequenced in MiSeq Illumina sequencer in the paired-end 300bp reads mode. The BCR repertoires were analyzed by R-package. Full-length sequences were annotated using the built-in murine reference alleles in MiXCR under default parameters and subsequently were exported by the VDJ region and CDR3 gene feature for clonotyping (v 3.0.1). Subsequent analysis was performed with R version 4.0.3.

### Statistics Analysis

The significance analysis was performed in GraphPad PRISM 6.0 (GraphPad Software, Inc. La Jolla, CA, USA). And p value from unpaired t-test was indicated as ≤0.05 (*), ≤0.01 (**), ≤0.001 (***), ≤0.0001 (****). All error bars were displayed as mean ± SEM.

## Results

### Accelerated Germinal Center (GC) Reactions in the Presence of TLR7 Signaling

**Figure 1:**
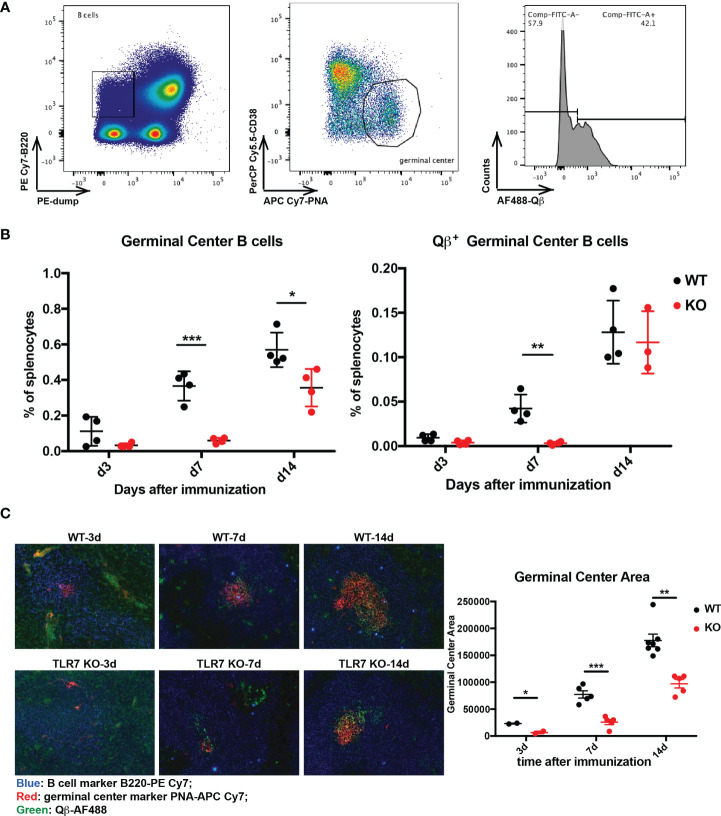
Examination of Qβ-specific germinal center reaction. **(A)** gating strategy to measure germinal center B cells and Qβ-specific cells by flow cytometry. B cells were defined as B220^high^ dump (CD4, CD8, IgM, IgD, CD11b, CD11c, GR1)^low^, from which PNA^high^ CD38^low^ germinal center cells were selected. Then Qβ^high^ cells from germinal center cells were marked. **(B)** Percentage of germinal center cells (left) and Qβ^+^ germinal center B cells (right) in splenocytes were plotted (n = 4). **(C)** Immuno-histology of Qβ-specific germinal centers of spleen sections from WT and TLR7 KO mice during 14 days after Qβ immunization. B cells were labeled as B220 (blue), germinal center as PNA (red) and Qβ (green). Analysis of germinal center areas with Image J 1.51s. p value from unpaired t-test was indicated as ≤0.05 (*), ≤0.01 (**), ≤0.001 (***). The figure is representative of two independent experiments.

GC reactions are critical to generate high affinity antibodies and to permit differentiation of fully differentiated long-lived plasma and memory B cells. Therefore, we first examined the effects of TLR7 signaling on GC reactions. To this end, we immunized wild type (WT) and TLR7 KO mice with RNA-loaded Qβ and harvested spleens at d3, d7, and d14 after immunization, followed by analysis by flow cytometry and immunohistochemistry. The GC B cells were characterized as PNA^+^ CD38^-^, among which Qβ^+^ cells were identified by fluorescence-labeled Qβ staining (**Figure 1A**). As shown in **Figure 1B**, Qβ-specific GC B cells of TLR7 KO mice were strongly reduced in numbers compared to WT mice at the early timepoint d7 but numbers caught up by d14, suggesting that lack of TLR7 delayed but did not abolish a GC reaction producing Qβ-specific B cells. Furthermore, histological analysis demonstrated that the area of GCs in WT mice was larger than in TLR7 KO mice (**Figure 1C**), and an increase in Qβ-specific cells was observed (shown as green). Taken together, deficiency of TLR7 reduced GC reactions at early stage, indicating the TLR7 signaling is of importance for antibody maturation, particular in early responses.

### Enhanced IgG Responses by TLR7 Signaling Upon VLP Immunization

**Figure 2:**
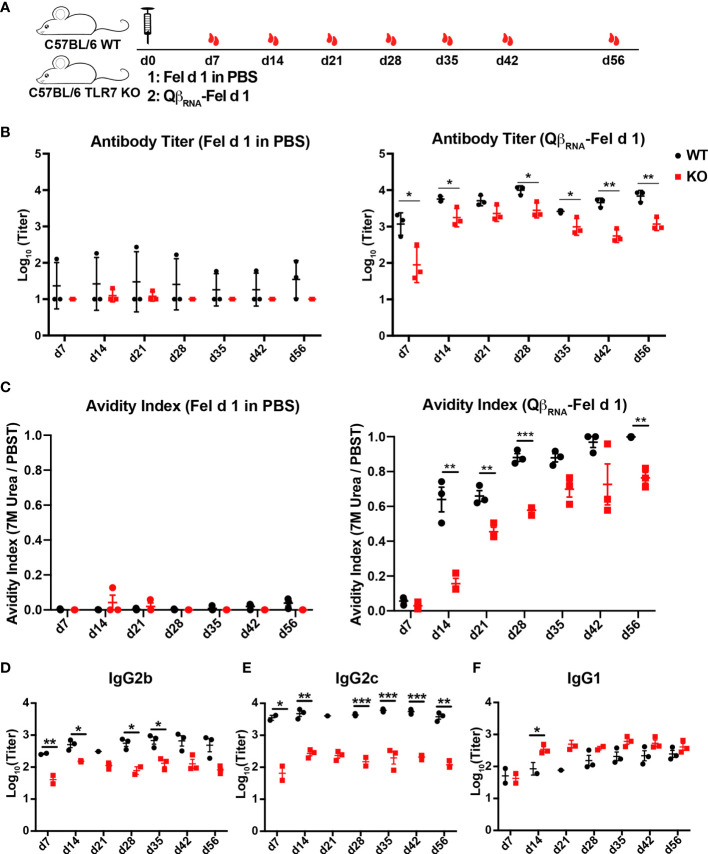
Fel d 1-specific antibody responses of WT and TLR7 KO mice upon Qβ_RNA_-Fel d 1 immunization. **(A)** Immunization scheme. three mice per group were injected with 30 μg Qβ_RNA_-Fel d 1 or Fel d 1 in PBS at d0, and mice were bled at d7, 14, 21, 28, 35, 42 and 56. Fel d 1-specific antibody titers **(B)** and avidity indexes **(C)** in WT and TRL7 KO mice after immunization were demonstrated. IgG subclasses 2b **(D)**, 2c **(E)** and 1 **(F)** in WT and TRL7 KO mice after Qβ_RNA_-Fel d 1 immunization were determined by ELISA. p value from unpaired t-test was indicated as ≤0.05 (*), ≤0.01 (**), ≤0.001 (***). The figure is representative of two independent experiments.

We next assessed the influence of TLR7 signaling deficiency on the antibody responses to Fel d 1 displayed on VLPs. WT and TLR7 KO mice were immunized with Qβ-Fel d 1 and serum samples were collected as indicated in **Figure 2A**. Soluble Fel d 1 in PBS was used as control. Fel d 1 specific IgG was significantly reduced in TLR7 KO mice compared to WT mice as shown in **Figure 2B**, suggesting that the TLR7 signaling was important to drive Fel d 1 specific IgG responses. In contrast, Fel d 1 in PBS failed to elicit measurable antibody responses in presence and absence of TLR7. In addition to antibody quantities, the quality of Fel d 1-specific antibodies was assessed by avidity ELISA. Because 7 M urea can wash away the low-avidity antibodies, avidity index was determined by the ratio of ELISA readout with 7 M urea wash to that with PBST wash. As shown in **Figure 2C**, avidity indexes of WT mice sera against Fel d 1 were dramatically higher than those of TLR7 KO mice, indicating that TLR7 facilitates generation of high affinity antibodies. Thus, TLR7 signaling contributes to better antibody quality and quantity.

**Figure 3:**
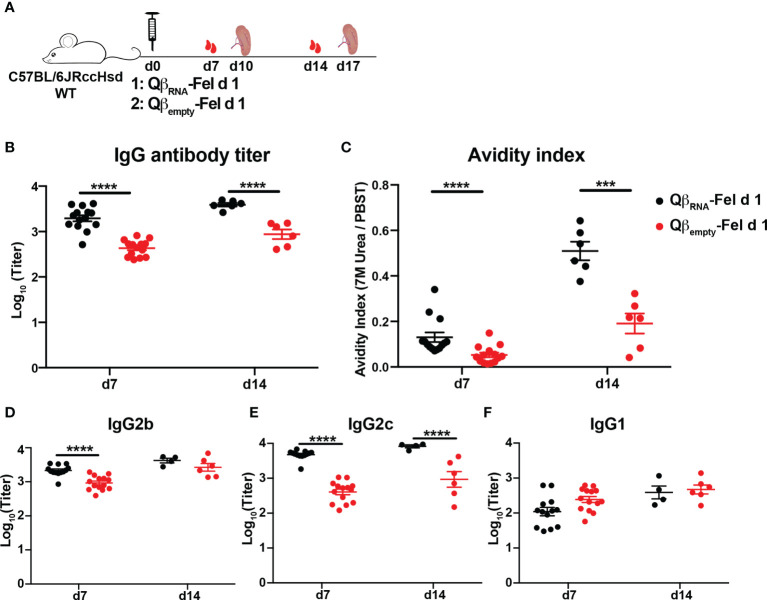
Fel d 1-specific antibody responses of WT mice upon Qβ_RNA_-Fel d 1 or Qβ_empty_-Fel d 1 immunization. **(A)** Immunization scheme. Fourteen mice were immunized with 30 μg Qβ_RNA_-Fel d 1 or Qβ_empty_-Fel d 1 at d0 and were bled at d7. Eight mice were euthanized to harvest spleen and sort Fel d 1-specific B cells at d10, so 6 mice were bled at d14 and euthanized for Fel d 1-specific B cells sorting at d17. Fel d 1-specific antibody titers **(B)**, avidity indexes **(C)**, and IgG2b **(D)**, IgG2c **(E)** and IgG1 **(F)** subclasses generated by WT mice after immunization were determined by ELISA. p value from unpaired t-test was indicated as ≤0.001 (***), ≤0.0001 (****). The figure is based on one experiment.

To confirm the importance of the ssRNA in the applied Qβ-VLPs, we next immunized WT mice with Fel d 1 conjugated to ssRNA-containing VLPs (Qβ_RNA_-Fel d 1) or ssRNA-deficient VLPs [Qβ_empty_-Fel d 1, in which ssRNA was removed by means of RNAse digestion (**Figure S1**) (20)]. In agreement with the above findings in TLR7-defecient mice, Fel d 1-specific antibody titers (**Figure 3B**) and avidity indexes (**Figure 3C**) induced by Qβ_RNA_-Fel d were significantly higher compared to mice immunized with Qβ_empty_-Fel d 1, corroborating the importance of RNA-induced signaling to promote antibody responses upon VLP immunization.

### Fel d 1-Specific IgG Antibodies Were Dominated by IgG2b and IgG2c Isotypes Compared to IgG1 Isotype in the Presence of TLR7 Signaling

TLR7 signaling was demonstrated previously to affect isotype switching induced by Qβ (17, 20). To address whether this was also the case for antigen displayed by Qβ; Fel d 1-specific IgG1, 2b and 2c antibodies were measured in sera from Qβ_RNA_-Fel d 1 immunized WT and TLR7 KO mice. ELISA results showed that Fel d 1-specific IgG2b and 2c antibodies were significantly reduced in TLR7 KO mice compared to those in WT mice (**Figure 2D, E**). In contrast, IgG1 antibody titers were higher in TLR7 KO mice than WT mice (**Figure 2F**), indicating that the TLR7 signaling mediated antibody switching to IgG2b/2c isotypes at the expense of IgG1 isotype. Consistently, IgG2b/2c antibodies in WT mice that were immunized with Qβ_RNA_-Fel d 1 were drastically higher than in mice immunized with Qβ_empty_-Fel d 1 (**Figures 3D, E**). IgG1 antibody titers in Qβ_empty_-Fel d 1 immunized mice were also slightly decreased (**Figure 3F**). Taken together, similar to TLR9 signaling (15, 34), TLR7 signaling triggered IgG2b/2c isotype switch and suppressed IgG1 production.

### TLR7 Intrinsic Signaling in B Cells Enhanced Anti-Fel d 1 IgG Antibody Responses

**Figure 4:**
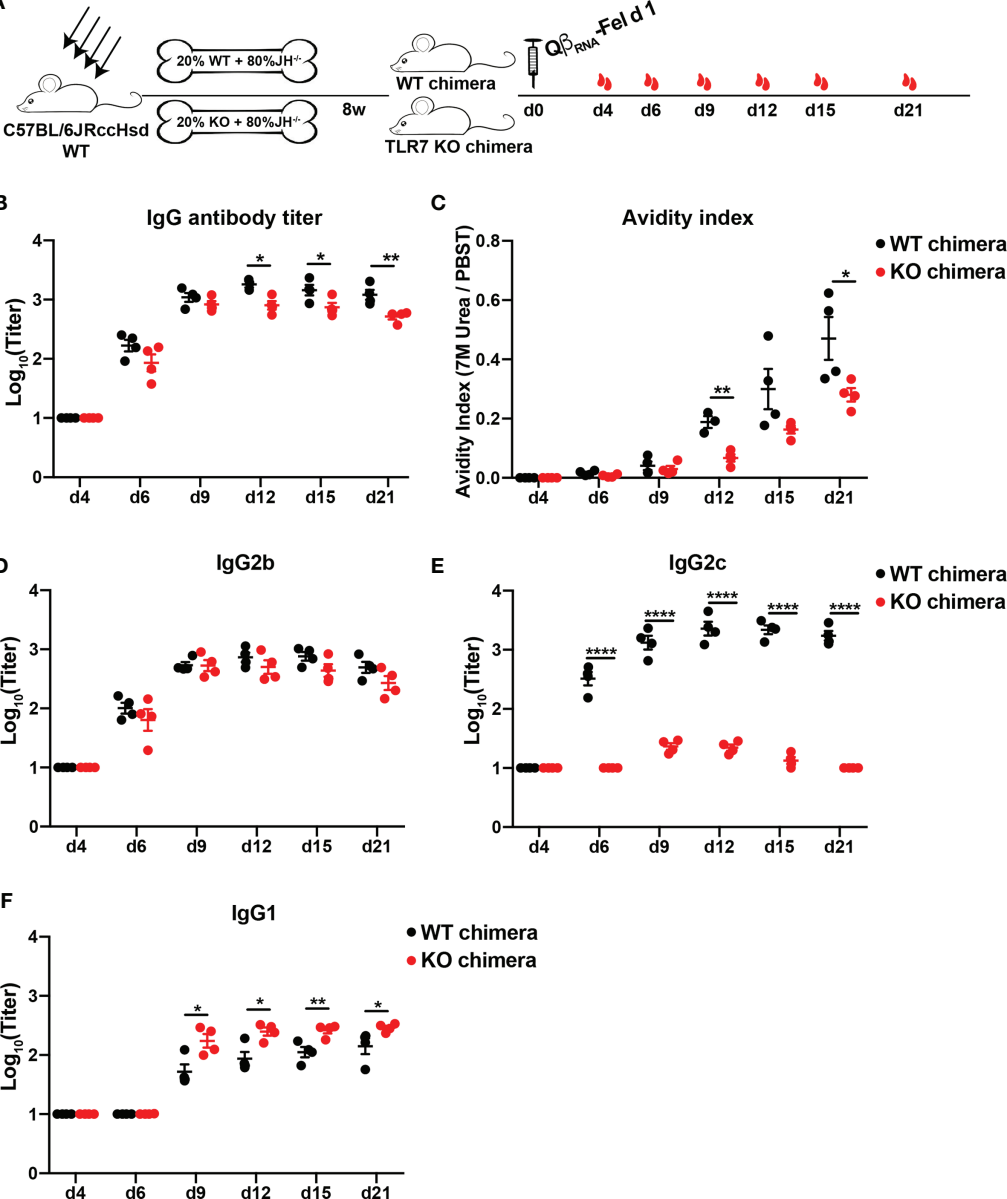
Antibody responses of bone marrow chimeric mice against Fel d 1 after Qβ_RNA_-Fel d 1 immunization. **(A)** Immunization scheme. Irradiated mice were reconstituted with 80% JH KO and 20% WT or 20% TLR7 KO BM cells. Three mice per group were injected subcutaneously with 30 μg Qβ_RNA_-Fel d 1 at d0, and mice were bled at d4, 6, 9, 12, 15, and 21. Total Fel d 1-specific IgG antibody titers **(B)**, avidity indexes **(C)** and IgG2b **(D)**, IgG2c **(E)** and IgG1 **(F)** antibody titers of WT and KO chimeras after Qβ_RNA_-Fel d 1 immunization were determined by ELISA. p value from unpaired t-test was indicated as ≤0.05 (*), ≤0.01 (**), ≤ 0.0001 (****). The figure is representative of two independent experiments.

To examine the role of B cell intrinsic TLR7 signaling in Fel d 1-specific antibody responses, TLR7 KO bone marrow (BM) chimeric mice (80% JH KO and 20% TLR7 KO BM cells) were generated and immunized with Qβ_RNA_-Fel d 1 (**Figure 4A**). As control, wild type BM chimeras were treated in the same way. Fel d 1-specific IgG antibodies in WT chimeras were remarkably higher than those in TLR7 KO chimeras (**Figure 4B**). In addition, high-avidity Fel d 1-specific IgG antibodies were drastically reduced in the absence of TLR7 signaling in B cells (**Figure 4C**). Slightly different from findings in TLR7 KO mice, TLR7 KO BM chimeric mice showed comparable IgG2b levels as WT chimeras, while IgG2c was nearly abolished in TLR7 KO chimeras (**Figures 4D, E**), implying the IgG2c isotype switch was strictly dependent on B cell intrinsic TLR7 signaling. In addition, IgG1 in TLR7 KO chimeras was significantly increased compared to WT chimeras (**Figure 4F**). These results demonstrate that TLR7 signaling in B cells is key to trigger IgG2b/2c isotype switch and to suppress class-switch to IgG1.

### TLR7 Signaling Drives and Maintains the Fel d 1-Specific B Cell Receptor (BCR) Repertoire at Higher Diversity

Due to the importance of TLR7 signaling in antibody responses, both in terms of quality and quantity after Qβ-Fel d 1 immunization, we investigated the BCR repertoire of Fel d 1-specific B cells. To collect Fel d 1-specific B cells, spleens of mice immunized with Qβ_RNA_-Fel d 1 or Qβ_empty_-Fel d 1 were harvested (10 and 17 days after immunization as shown in **Figure 3A**) and processed to allow sorting of Fel d 1-specific B cells by FACS. The sorted Fel d 1-specific B cell counts from Qβ_RNA_-Fel d 1 immunized mice were 10-fold more than those from Qβ_empty_-Fel d 1 immunized mice (**Table S2**), confirming that lack of TLR7 signaling impaired Fel d 1-specific B cell responses. Nevertheless, variable regions of IgG1, 2b and 2c could be amplified from cDNA of all samples (d10_Qβ_RNA_-Fel d 1, d10_Qβ_empty_-Fel d 1, d17_Qβ_RNA_-Fel d 1, d17_Qβ_empty_-Fel d 1). No IgG2c amplicons could be obtained from the d10_Qβ_empty_-Fel d 1 sample, indicating that the sorted cells were dominated by IgG1 and IgG2b. Accordingly, there were more CDR3 and VDJ clonotypes for IgG1, 2b and 2c of RNA-adjuvanted immunized group (**Table S3**).

**Figure 5:**
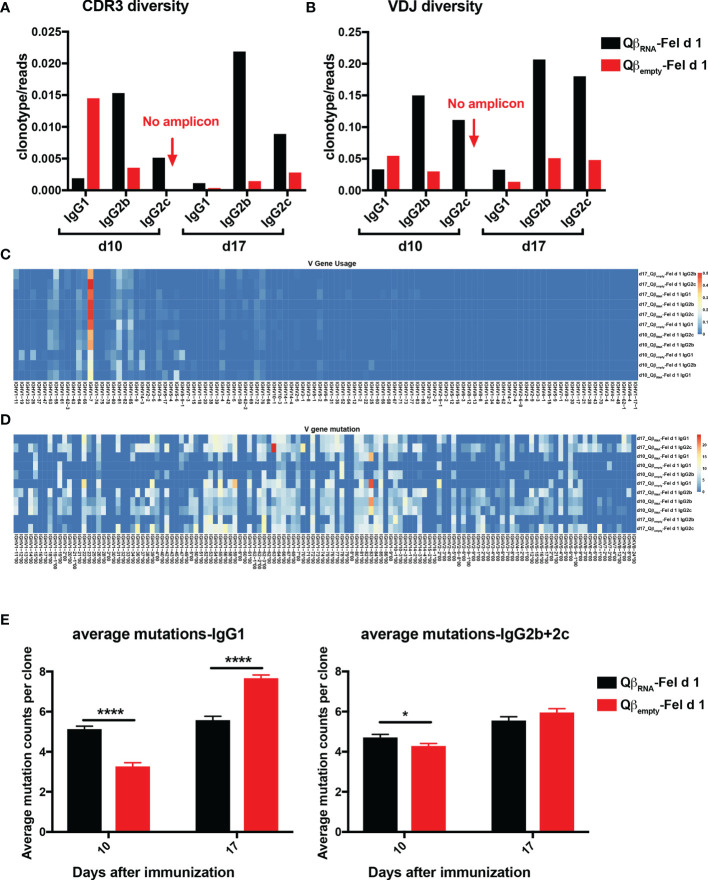
Fel d 1-specific IgG BCR repertoires from Qβ_RNA_-Fel d 1 and Qβ_empty_-Fel d 1 immunized mice. **(A, B)** Diversity of IgG1, 2b, 2c repertoires in terms of CDR3 sequences **(A)** and VDJ combinations **(B)** at d10 and d17 after Qβ_RNA_-Fel d 1 and Qβ_empty_-Fel d 1 immunization. Diversity of each repertoire was determined as the clonotype counts normalized by read counts. **(C)** Frequency of IGHV gene usage by different repertoires. **(D)** Mutation frequency on germline IGHV sequences in Fel d 1-specific BCR repertoires; **(E)** Average mutations of IgG1 and IgG2b+2c repertoires at different time points after Qβ_RNA_-Fel d 1 and Qβ_empty_-Fel d 1 immunization. p value from unpaired t-test was indicated as ≤0.05 (*), ≤0.001 (***). The figure is representative of two independent experiments.

The diversity of the respective BCR repertoires were determined by normalizing the clonotypes with sequencing reads. IgG2b and 2c exhibited a higher degree of clonal diversity than IgG1 in Qβ_RNA_-Fel d 1 immunized mice at both d10 and d17 after immunization in terms of unique CDR3 amino acid (AA) sequences (**Figure 5A**) as well as when clonotyping by full-length VDJ region (**Figure 5B**). In addition, IgG1 clonotypes of d10_Qβ_empty_-Fel d 1 showed higher diversity than d10_Qβ_RNA_-Fel d 1. In contrast, at the d17 time point, IgG1 antibodies induced in presence of RNA showed slightly higher VDJ diversity, indicating that IgG1 subclass diversity is not affected by absence of TLR7 signaling. Importantly, the CDR3- and VDJ-diversity increased between day 10 and 17 in presence of TLR7 signaling, whereas unexpectedly, the diversity strongly decreased in that timeframe in the absence of TLR7 signaling. This demonstrates that TLR7 signaling is responsible for early induction and maintaining high clonal diversity.

Among all VDJ clonotypes, IGHV1-7 gene was noted to be dominant in all Fel d 1-specific B cells, regardless of TLR7 signaling or time after immunization (**Figure 5C**), which coincidently is also the V_H_ gene element used by one of the previously identified Fel d 1-specific monoclonal antibodies A044 (29). The distribution of average mutations in each V-gene family was not biased (**Figure 5D**), which contributed to the diversity of BCR repertoires. In addition, the average mutation counts of IgG1 and IgG2b repertoire at d10 in both Qβ_RNA_-Fel d 1 and Qβ_empty_-Fel d 1 immunization were less than at d17 (**Figure 5E**), indicating the somatic hypermutation (SHM) was progressing after day 10. Thus, the data demonstrate an important function of TLR7 signaling to generate BCR-diversity early or shortly after VDJ-rearrangement rather than to keep hypermutation at a high rate. Furthermore, the IgG1 and 2b/2c mutations in d10_Qβ_RNA_-Fel d 1 were higher than d10_Qβ_empty_-Fel d 1 pool, whereas the opposite was observed on d17 after immunization. This paradox that higher average mutation counts occurred in the absence of TLR7 signaling could be attributed to the enlarged number of clonotypes on d17 after Qβ_RNA_-Fel d 1 immunization. Overall, these data unexpectedly suggest that TLR7-signaling impacts BCR-diversity more strongly than BCR mutation rates.

### Fel d 1-Specific BCR Repertoire Are Closely Related, in Particular for IgG2b and 2c

**Figure 6:**
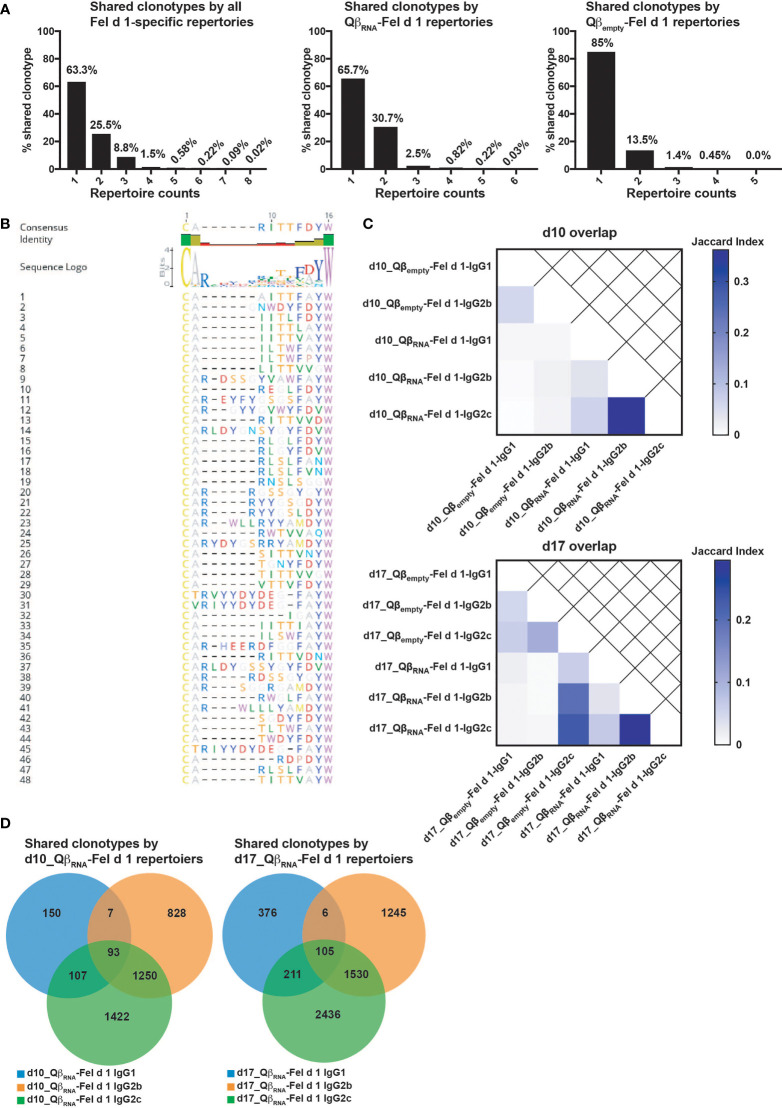
Shared CDR3 clonotypes among Fel d 1-specific BCR repertoires. **(A)** Percentage of CDR3 sequences shared by different samples of all obtained BCR repertories, RNA-adjuvanted repertoires and non-RNA adjuvanted repertoires; **(B)** Alignment of top 48 CDR3 sequences that shared by more than 6 samples. The consensus sequence was acquired by Geneious Prime; **(C)** Jaccard indexes of overlapped CDR3 clonotypes among BCR repertoires at d10 or d17 after immunization; **(D)** Venn-diagrams of shared CDR3 clonotypes among IgG isotype repertoires at d10 or d17 after Qβ_RNA_-Fel d 1 immunization. The figure is representative of two independent experiments.

Despite the substantial variation of IgG repertoires diversity, it was noted that many CDR3 sequences were shared across different isotypes and mice regardless of time points (**Figure 6A**). In all IgG subclass repertoires at both time points, there were 36.7% of CDR3 sequences shared by at least 2 IgG subclass repertoires. More specifically, there were 34.3% of CDR3 sequences shared by over 2 samples immunized with Qβ_RNA_-Fel d 1, while only 15% were shared in Qβ_empty_-Fel d 1 immunized repertoires. Strikingly, there were 48 CDR3 sequences shared by most Qβ_RNA_-Fel d 1 immunized repertoires at both time points, among which 28 out of 48 CDR3 sequences had a length of 10 AA. To reveal potential conservation of CDR3 sequences for Fel d 1-specificity, alignment of these sequences was performed. As shown in **Figure 6B**, AA were relatively conserved at both ends of CDR3 sequences, especially the C terminus. These findings imply relations between repertoires specific for Fel d 1 both in presence and absence of TLR7 signaling, indicating that Fel d 1 specific antibodies have a partially public character.

To further study the impacts of TLR7 signaling on the selection of CDR3 sequences into the pool of specific antibodies, the overlapping clonotypes were analyzed pairwise in Qβ_empty_-Fel d 1 and Qβ_RNA_-Fel d 1 immunized repertoires, respectively. **Figure 6C** illustrates that IgG2b and 2c were more closely related to each other than to IgG1. On the other hand, IgG repertoires were poorly comparable at different time points after immunization due to their origin in different mice. In this sense, IgG isotypes at the same time point displayed more overlapping CDR3 sequences than comparing repertoires at different time points (i.e. different mice as well). Importantly, the overlap among IgG subclasses in Qβ_RNA_-Fel d 1 immunization repertoires was more pronounced than Qβ_empty_-Fel d 1 immunizations, suggesting the TLR7 signaling facilitated the similarities of Fel d 1-specific IgG isotypes, perhaps due to facilitation of earlier expansion of fewer clones. To better understand the relations between repertoires, Venn-diagrams were created (**Figure 6D**). Consistently, IgG2b and 2c shared the most CDR3 sequences in both d10 and d17_Qβ_RNA_-Fel d 1 immunization repertoires (1250, 1530 respectively). There were similar counts of sequence shared by all 3 isotypes at d10 (93) and d17 (105) after immunization, indicating that clonal selection occurred early while mutation and maturation of each isotypes continued after d10 individually.

**Figure 7:**
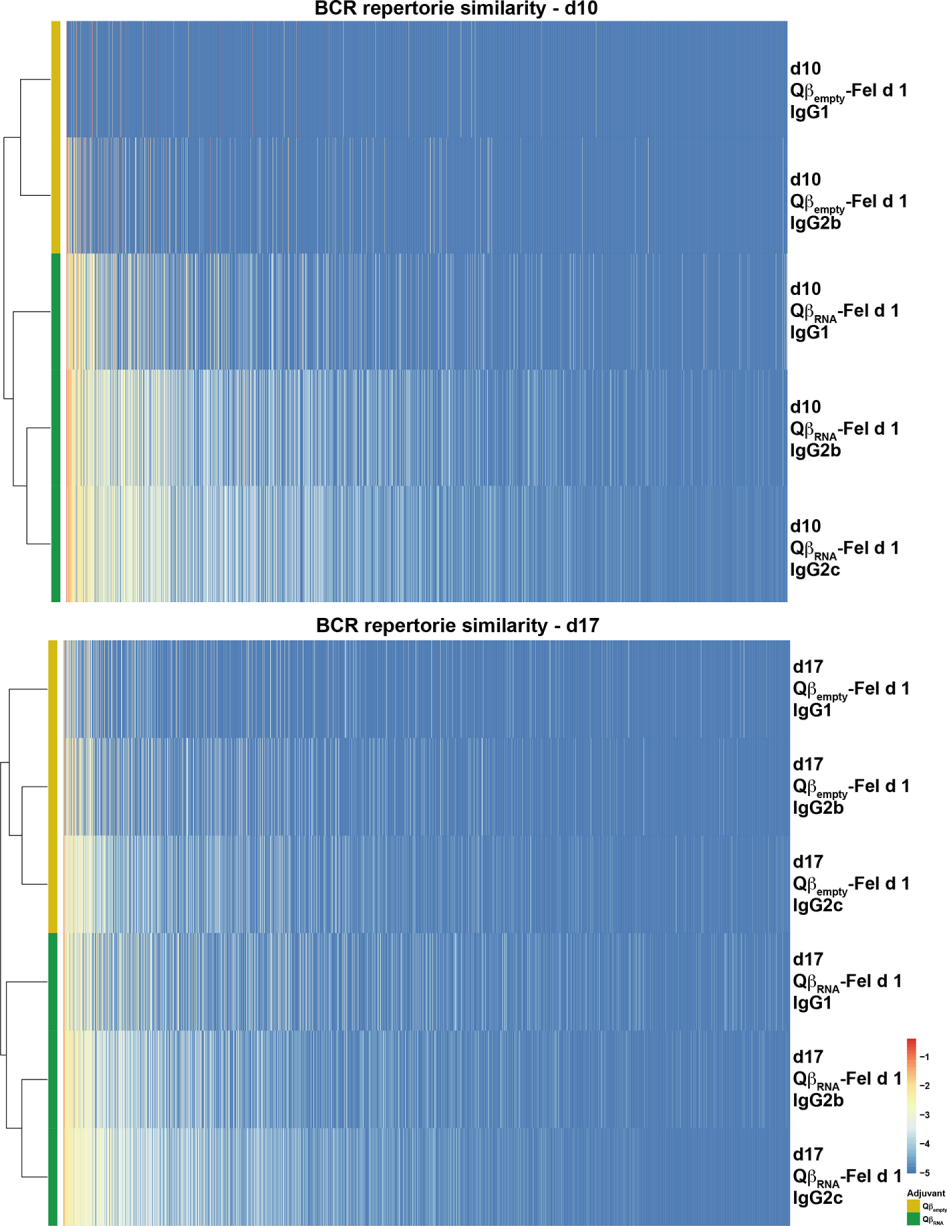
Similarity of Fel d 1-specific IgG BCR repertories at d10 and d17 after immunization. The CDR3 clonotypes were displayed, usage frequency was shown as each colored bar, and the phylogenetic tree was created according to the overall CDR3 sequence similarities among each repertoire. The figure is representative of two independent experiments.

To further analyze BCR development, the distribution of CDR3 sequences of Fel d 1-specific BCR repertoires were displayed as phylogenetic trees, illustrating that the IgG repertoires with TLR7 signaling were isolated from repertoires without TLR7 signaling at both d10 and d17 after immunization (**Figure 7**). Again, the distance between IgG2b and 2c was smaller than the distance observed for IgG1.

## Discussion

VLPs that are produced in *E. coli* have practical advantages, like high yield and low production costs. VLPs may not only be used to immunize against the virus they originate from but can also be used as “display-platforms” to immunize against antigens of choice attached to their surface. Such vaccines are mostly made in two different ways, namely chemical coupling or genetic fusion (2). Chemical coupling may be used for rapid testing of vaccine candidates due to its straightforward and quick access, and more importantly natural antigen conformation. On the other hand, genetic fusion is rather preferred in terms of large-scale application as it may result in stable and economic production. Nevertheless, both methods involve RNA packaging from the producer cell line and TLR7 signaling. Therefore, it is meaningful to determine the contribution of TLR7 signaling to the immunogenicity of VLP-based vaccines.

The current work investigated in depth the influence of TLR7 signaling on antibody responses against Fel d 1, displayed on the surface of Qβ VLPs. B cell intrinsic TLR7 signaling has previously been shown to drive Qβ-specific IgG antibody responses of higher quantity and quality (19), and this finding is now extended to Fel d 1 displayed by the VLP. Furthermore, isotype-specific ELISA and BCR repertoire analysis both demonstrated that IgG2b and 2c were preferred rather than IgG1 in presence of TLR7 signaling. Generally, Fel d 1-specific BCR repertoires indicated that IgG2b and 2c shared closer clonal relations, and more diversity in the presence of TLR7 signaling, which could explain the higher avidity antibodies observed in the presence of TLR7 signaling due to selection of more diverse antibodies. Lastly, the VDJ clonotypes of Fel d 1-specific BCRs were biased and CDR3 sequences were slightly conserved, indicating a partially public character.

The role of TLR7 signaling in immunogenicity of Qβ as a model antigen has been investigated previously. For instance, it has been demonstrated that TLR7 signaling facilitates antibody generation and IgG2a/2b switch in mice after Qβ immunization (17). Importantly, we have recently shown that TLR7 signaling licensed generation of secondary plasma cells that generate rapidly high levels of antibodies but are short-lived (19). Here we demonstrate that TLR7 signaling is essential for GC reactions after Qβ immunization, which was consistent with reduced avidity of antibodies in the absence of TLR7 signaling. Moreover, lack of RNA ligand and TLR7 receptor simultaneously impaired the Fel d 1-specific antibody titers and avidity indexes, corroborating that TLR7 signaling is important for antibody maturation after VLP immunization. In particular, TLR7 deficiency specifically in B cells was the main cause of the impaired responses, demonstrating that TLR7 drives antibody responses in a B cell intrinsic fashion. It is worth to note that the IgG2b titers were not influenced by lack of TLR7 in B cells; instead, the IgG2c response was basically erased, indicating the IgG2c switch was most strongly dependent on TLR7 signaling in B cells. We have previously shown similar findings for TLR9 signaling, where IgG2a/c antibody production was also driven by a B cell intrinsic mechanism (15, 34). In summary, TLRs in B cells directly sense ligands and drive antibody to IgG2a/2c switch.

Comparison of BCR repertoires demonstrated that TLR7 signaling overall enhanced antibody diversity and to a lower degree, somatic hypermutation. The sorted Fel d 1-specific cell counts were much higher in the presence of RNA, and the clonotype counts in RNA-adjuvanted samples exceeded the samples of the non-RNA group. However, clonotype counts may not represent diversity but rather indicate clonal expansion of Fel d 1-specific B cells. It is therefore important to note that IgGs of RNA-adjuvanted samples also showed higher normalized diversity than non-RNA adjuvanted ones, with the exception that lower IgG1 diversity at d10, corresponding to the higher IgG1 titer induced in the absence of TLR7 signaling. More interestingly, TLR7 signaling not only induced high diversity early on but was also responsible for the maintenance of diversity. Mutations in V_H_ genes showed a somewhat conflicting pattern for IgG1, as more mutations were found in the presence of RNA at d10 while less were found at d17. However, this might be due to the limited sample size after Qβ_empty_-Fel d 1 immunization. Taking into account that Qβ induced heterogenous antibody repertoires in marginal zone (MZ) B cells (35), there could be some Fel d 1-specific B cells in MZ rather than GC after Qβ_empty_-Fel d 1 immunization, leading to reasonable overall antibody response despite extremely low cellular counts in GCs, which should be investigated further.

It seems reasonable to speculate that the dominant IGHV1-7 gene is specific to Fel d 1 in germline configuration because of the predominant usage among all the V-gene fragments. In addition, we have shown that mature configuration of IGHV1-7 binds to Fel d 1 with high affinity (28). The germline configuration recognizes the same epitope of Fel d 1 as the mature counterpart with lower affinity, but was also able to activate mast cell degranulation in IgE format demonstrating functional activity of germline IGHV1-7 (30). Lastly, the high similarity and shared clonotypes of BCR repertoires among RNA-adjuvanted samples inferred the same origin of Fel d 1-specific antibodies, which may indicate that antibodies specific to certain antigen were homologous to some extent.

In summary, we show here that TLR7 signaling, especially in B cells, drives early GC formation and affinity-maturation and is responsible for induction and maintenance of BCR diversity.

## Data Availability Statement

The data presented in the study are deposited in the Zenodo repository, DOI: 10.5281/zenodo.5817898.

## Ethics Statement

The animal study was reviewed and approved by Cantonal Veterinary Office Bern, Switzerland.

## Author Contributions

XC wrote the manuscript. XC and PK performed most experiments and analyzed data. CK generated bone marrow chimeric mice. JH and AY analyzed BCR repertoire data. LZ, SR, MM, and AO helped reviewing the manuscript. MV and MFB designed and supervised the study and improved the writing. All authors contributed to the article and approved the submitted version.

## Funding

This work was sponsored by Swiss National Science Foundation grant to MFB (SNF Nr. 310030 185114) and a PhD fellowship from China Scholarship Council (CSC Nr. 201706740091, to XC).

## Conflict of Interest

MFB is involved in companies that develop vaccines based on virus-like particle.

The remaining authors declare that the research was conducted in the absence of any commercial or financial relationships that could be construed as a potential conflict of interest.

## Publisher’s Note

All claims expressed in this article are solely those of the authors and do not necessarily represent those of their affiliated organizations, or those of the publisher, the editors and the reviewers. Any product that may be evaluated in this article, or claim that may be made by its manufacturer, is not guaranteed or endorsed by the publisher.

## Supplementary Material

Supplementary Figure 1 Analysis of Qβ-Fel d 1 using agarose and SDS-PAGE gel. **(A)** Removal of RNA from Qβ. Lane 1: Qβ after RNase digestion; lane 2: Qβ before RNase digestion. **(B)** Chemical coupling of Fel d 1 to Qβ. Lane 1: Qβ linked to SMPH; lane 2: Fel d 1 conjugated to Qβ; lane 3: Fel d 1, and the Qβ-Fel d 1 band was indicated with star. The figure is representative of three independent experiments.

Supplementary Figure 2 Gating strategy to sort Fel d 1-specific germinal center B cells. From left to right: mature B cells (B220^+^IgD^-^IgM^-^), germinal center B cells (PNA^+^CD38^-^), Fel d 1-specific germinal center B cells (Fel d 1^+^AP205-Fel d 1^+^).
