# Supplementary figures and images for: TLR7 Signaling Shapes and Maintains Antibody Diversity Upon Virus-Like Particle Immunization
Source: Front Immunol. 2022 Jan 19;12:827256. doi: 10.3389/fimmu.2021.827256 (PMC8807482; doi:10.3389/fimmu.2021.827256)

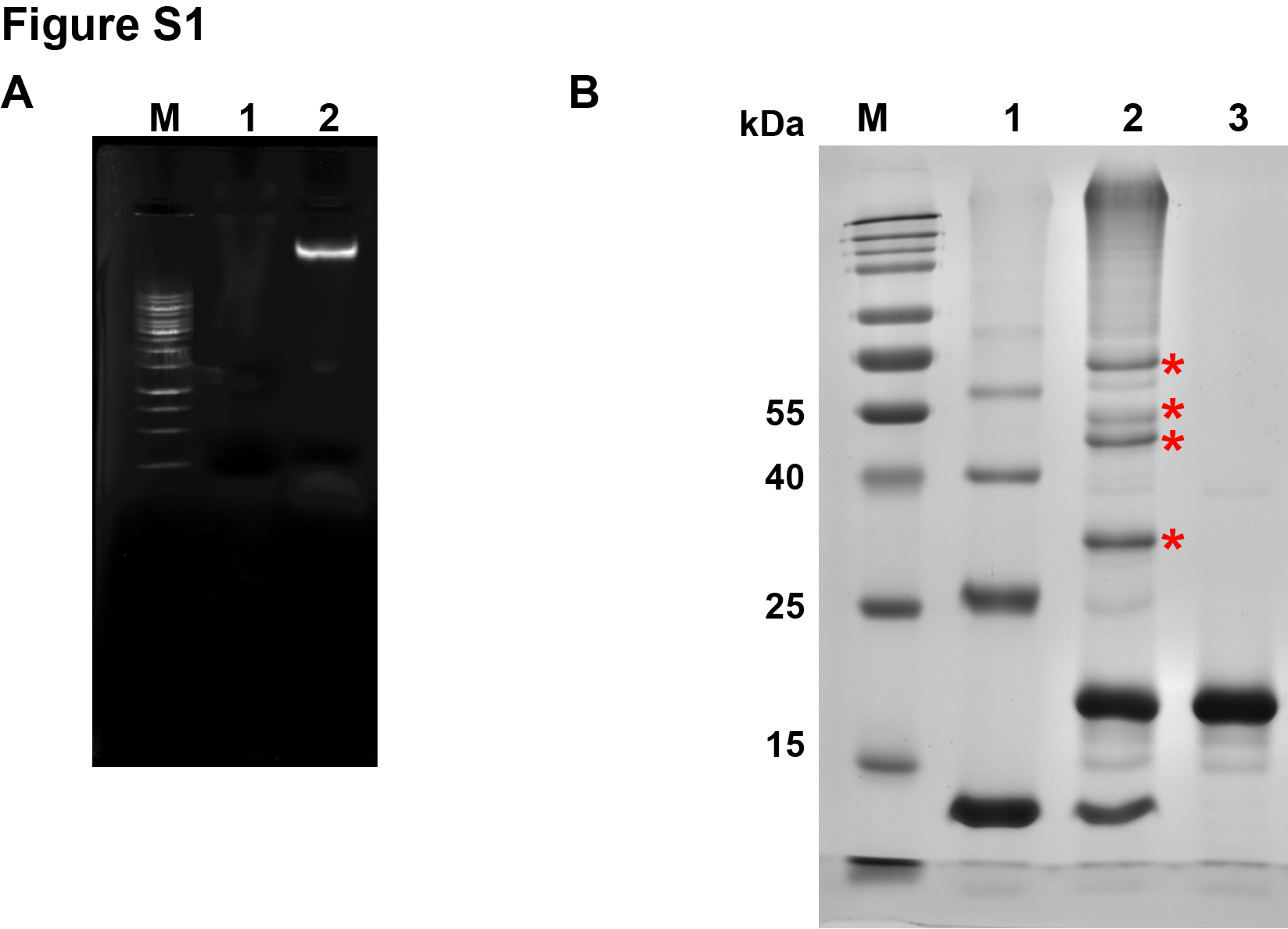

Supplement: Supplementary Figure 1 — Analysis of Qβ-Fel d 1 using agarose and SDS-PAGE gel. (A) Removal of RNA from Qβ. Lane 1: Qβ after RNase digestion; lane 2: Qβ before RNase digestion. (B) Chemical coupling of Fel d 1 to Qβ. Lane 1: Qβ linked to SMPH; lane 2: Fel d 1 conjugated to Qβ; lane 3: Fel d 1, and the Qβ-Fel d 1 band was indicated with star. The figure is representative of three independent experiments. [file Image_1.jpeg]

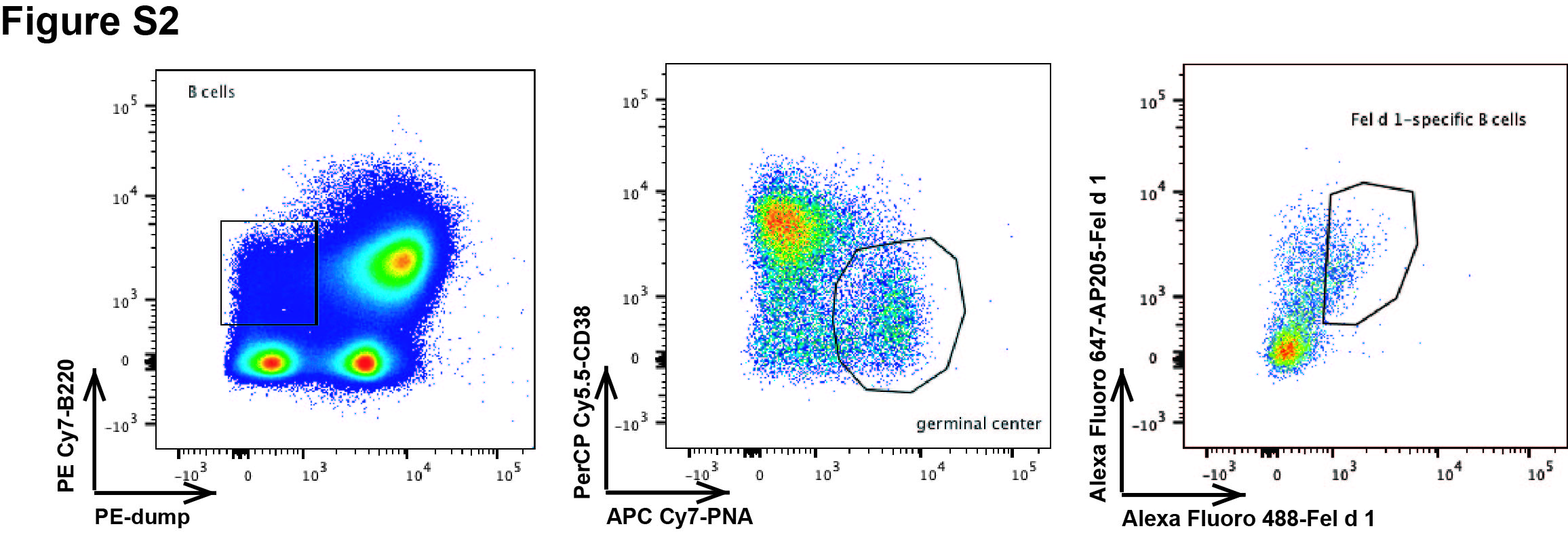

Supplement: Supplementary Figure 2 — Gating strategy to sort Fel d 1-specific germinal center B cells. From left to right: mature B cells (B220+IgD-IgM-), germinal center B cells (PNA+CD38-), Fel d 1-specific germinal center B cells (Fel d 1+AP205-Fel d 1+). [file Image_2.jpeg]
