# Supplementary material for: TLR7 Signaling Shapes and Maintains Antibody Diversity Upon Virus-Like Particle Immunization
Source: Front Immunol. 2022 Jan 19;12:827256. doi: 10.3389/fimmu.2021.827256 (PMC8807482; doi:10.3389/fimmu.2021.827256)
Supplement: Supplementary file 3 [file Table_1.docx]

**Table S1.** Sequences of primer for VH fragment amplification

| Primer | DNA sequence (5’to 3’) | Length (bp) |
| --- | --- | --- |
| IgH-fw1 | TCGTCGGCAGCGTCAGATGTGTATAAGAGACAGNNNNGAKGTRMAGCTTCAGGAGTC | 57 |
| IgH-fw2 | TCGTCGGCAGCGTCAGATGTGTATAAGAGACAGNNNNGAGGTBCAGCTBCAGCAGTC | 57 |
| IgH-fw3 | TCGTCGGCAGCGTCAGATGTGTATAAGAGACAGNNNNCAGGTGCAGCTGAAGSASTC | 57 |
| IgH-fw4 | TCGTCGGCAGCGTCAGATGTGTATAAGAGACAGNNNNGAGGTCCARCTGCAACARTC | 57 |
| IgH-fw5 | TCGTCGGCAGCGTCAGATGTGTATAAGAGACAGNNNNCAGGTYCAGCTBCAGCARTC | 57 |
| IgH-fw6 | TCGTCGGCAGCGTCAGATGTGTATAAGAGACAGNNNNCAGGTYCARCTGCAGCAGTC | 57 |
| IgH-fw7 | TCGTCGGCAGCGTCAGATGTGTATAAGAGACAGNNNNCAGGTCCACGTGAAGCAGTC | 57 |
| IgH-fw8 | TCGTCGGCAGCGTCAGATGTGTATAAGAGACAGNNNNGAGGTGAASSTGGTGGAATC | 57 |
| IgH-fw9 | TCGTCGGCAGCGTCAGATGTGTATAAGAGACAGNNNNGAVGTGAWGYTGGTGGAGTC | 57 |
| IgH-fw10 | TCGTCGGCAGCGTCAGATGTGTATAAGAGACAGNNNNGAGGTGCAGSKGGTGGAGTC | 57 |
| IgH-fw11 | TCGTCGGCAGCGTCAGATGTGTATAAGAGACAGNNNNGAKGTGCAMCTGGTGGAGTC | 57 |
| IgH-fw12 | TCGTCGGCAGCGTCAGATGTGTATAAGAGACAGNNNNGAGGTGAAGCTGATGGARTC | 57 |
| IgH-fw13 | TCGTCGGCAGCGTCAGATGTGTATAAGAGACAGNNNNGAGGTGCARCTTGTTGAGTC | 57 |
| IgH-fw14 | TCGTCGGCAGCGTCAGATGTGTATAAGAGACAGNNNNGARGTRAAGCTTCTCGAGTC | 57 |
| IgH-fw15 | TCGTCGGCAGCGTCAGATGTGTATAAGAGACAGNNNNGAAGTGAARSTTGAGGAGTC | 57 |
| IgH-fw16 | TCGTCGGCAGCGTCAGATGTGTATAAGAGACAGNNNNCAGGTTACTCTRAAAGWGTSTG | 59 |
| IgH-fw17 | TCGTCGGCAGCGTCAGATGTGTATAAGAGACAGNNNNCAGGTCCAACTVCAGCARCC | 57 |
| IgH-fw18 | TCGTCGGCAGCGTCAGATGTGTATAAGAGACAGNNNNGATGTGAACTTGGAAGTGTC | 57 |
| IgH-fw19 | TCGTCGGCAGCGTCAGATGTGTATAAGAGACAGNNNNGAGGTGAAGGTCATCGAGTC | 57 |
| IgG1_Rev | GTCTCGTGGGCTCGGAGATGTGTATAAGAGACAGNNNNATGGAGTTAGTTTGGGCAGCA | 59 |
| IgG2b_Rev | GTCTCGTGGGCTCGGAGATGTGTATAAGAGACAGNNNNTTGTATCTCCACACCCAGGG | 58 |
| IgG2c_Rev | GTCTCGTGGGCTCGGAGATGTGTATAAGAGACAGNNNNGTACCTCCACACACAGGGGCCAGTGGATAG | 68 |

**Table S2.** Sorted Fel d 1-specific B cell counts

| Days after immunization | Qβ_RNA_-Fel d 1 | Qβ_empty_-Fel d 1 |
| --- | --- | --- |
| d10 | 10684 | 1141 |
| d17 | 67531 | 2421 |

**Table S3.** Clonotype counts of BCR repertories

| Samples | VDJ clonotype | CDR3 clonotype |
| --- | --- | --- |
| d10_Qβ_RNA_-Fel d 1-IgG1 | 6651 | 382 |
| d10_Qβ_empty_-Fel d 1-IgG1 | 238 | 63 |
| d10_Qβ_RNA_-Fel d 1-IgG2b | 23867 | 2438 |
| d10_Qβ_empty_-Fel d 1-IgG2b | 1537 | 181 |
| d10_Qβ_RNA_-Fel d 1-IgG2c | 68627 | 3168 |
| d17_Qβ_RNA_-Fel d 1-IgG1 | 21983 | 761 |
| d17_Qβ_empty_-Fel d 1-IgG1 | 8962 | 222 |
| d17_Qβ_RNA_-Fel d 1-IgG2b | 33125 | 3506 |
| d17_Qβ_empty_-Fel d 1-IgG2b | 13395 | 381 |
| d17_Qβ_RNA_-Fel d 1-IgG2c | 102717 | 5062 |
| d17_Qβ_empty_-Fel d 1-IgG2c | 35073 | 2036 |
